# Characterization of primary small intestinal lymphoma: a retrospective study based on double balloon endoscopy

**DOI:** 10.1186/s12876-024-03193-z

**Published:** 2024-03-20

**Authors:** Lin Li, Huijian Ma, Meng Niu, Chunxiao Chen, Chaohui Yu, Hong Zhang, Meng Jin

**Affiliations:** Department of Gastroenterology, The First Affiliated Hospital, College of Medicine, Zhejiang University, Hangzhou, China

**Keywords:** Double balloon endoscopy, Endoscopic finding, Primary gastrointestinal lymphoma, Radiological finding

## Abstract

**Background:**

The diagnosis of primary small intestinal lymphoma (PSIL) is difficult. This study aimed to evaluate the clinical, radiological and endoscopic characteristics of PSIL and provide clue for diagnosis.

**Methods:**

A total of 30 patients diagnosed with PSIL who underwent double balloon endoscopy (DBE) in the First Affiliated Hospital of Zhejiang University were retrospectively analyzed. Clinical, radiological and endoscopic data were collected. Univariate analysis was used to determine significant indicators for differentiating three main subtypes of PSIL. Cox regression analysis was performed to assess the risk factors for survival.

**Results:**

In this study, 10 patients were pathologically diagnosed as diffuse large B-cell lymphoma (DLBCL), 11 were indolent B-cell lymphoma (BCL) and 9 were T-cell lymphoma (TCL). Compared with DLBCL patients, the body mass index (BMI) of TCL patients was significantly lower (*p* = 0.004). Meanwhile, compared with patients with DLBCL, the patients with indolent BCL had lower levels of C-reactive protein, lactate dehydrogenase (LDH), fibrinogen and D-Dimer (*p* = 0.004, *p* = 0.004, *p* = 0.006, and *p* = 0.002, respectively), and lower proportion of thicker intestinal wall and aneurysmal dilation in CT scan (*p* = 0.003 and *p* = 0.020, respectively). In terms of ulcer morphology, patients with DLBCL had significantly higher proportion of deep ulcers than patients with indolent BCL (*p* = 0.020, respectively). Cox regression analysis showed that drink (*p* = 0.034), concomitant colonic ulcers (*p* = 0.034) and elevated LDH (*p* = 0.043) are risk factors for mortality in patients with PSIL.

**Conclusions:**

This study provides clinical characteristics of patients with PSIL. Thicker intestinal wall and aneurismal dilation detected on CT scan and deeper ulcer on DBE examination helps to establish a diagnosis of DLBCL.

## Introduction

Lymphoma is a heterogeneous entity which includes Hodgkin’s lymphoma (HL) and non-Hodgkin’s lymphoma (NHL) [1]. The gastrointestinal tract is one of the most common organs that might be involved in extranodal lymphoma [2]. Primary gastrointestinal lymphoma (PGIL) originates from the submucosal lymphoid tissue of the gastrointestinal tract [3]. The lesions can be single or multiple in different parts of the digestive tract. The stomach is the most common site of PGIL, while primary small intestinal lymphoma (PSIL) is rare [4]. Though rare, there is a trend of gradual increase in the number of small intestinal lymphoma cases [5]. DLBCL is the earliest recognized intestinal lymphoma [6] and the most common intestinal lymphoma [3]. Numerous retrospective studies report the characters and outcomes of PGIL patients [7, 8], but there is little information about the difference between DLBCL with B- and T-cell lymphomas.

Previous cases reported that highly aggressive lymphoma is often accompanied by deep ulcers [9, 10]. Intestinal lymphoma often presents with aneurysmal dilation [11, 12]. In the past, PSIL was easily overlooked due to its veiled by a number of so-called “masks” of enteropathies, which is often diagnosed when patients present with intestinal perforation or obstruction. Moreover, the small intestine is difficult to reach by gastroscopy or colonoscopy and the pathology of biopsy is often false-negative, which lead to unnecessary surgical trauma [13]. The data on the modalities of small bowel endoscopy was still limited [14]. During the last decade, double balloon endoscopy (DBE) has been widely used as an examination and invention tool in the gastrointestinal tract [15], and has filled the gap in the diagnosis of small bowel lymphoma. Therefore, the diagnostic algorithm of PSIL needs to be updated.

In this study, we retrospectively investigated DBE features and biopsy findings in patients with PSIL, as well as their clinical features, treatment and outcomes.

## Methods

### Patients

From May 2013 to September 2021, 255 small intestinal biopsy examinations were performed in 255 patients admitted in the First Affiliated Hospital of Zhejiang University. 32 patients were pathologically diagnosed as PSIL. Clinical, radiological, and endoscopic data were collected. 2 patients were excluded because they were lost to follow-up. All cases were admitted in the First Affiliated Hospital of Zhejiang University. All biopsy samples were reviewed by the same pathologist (Jun Li). Imaging studies were independently reviewed by two abdominal imaging specialists (Baishu Zhong and LingxiangRuan) with more than 20 years of imaging differential diagnosis of digestive system diseases. For markings from the two radiologists that did not agree, each radiologist re-read the cases independently to assess the discordant markings. The mortality status was followed by telephone at the end of follow up date (2022/12/31). The median follows up period of the 30 cases was 21.5 months.

Demographic data were collected from the electronic medical record system. The resident admission records were completed within 24 h after admission. All demographic data showed in Table 1 were asked to be recorded according to electronic medical record system quality control. All information were provided by patient themselves or immediate family members. Cigarette smoking history was defined as regularly smoking before admission. Alcohol drinking was defined as regularly alcohol intake before admission. The history of cigarette smoking and alcohol drinking were provided by patient self-reporting.

**Table 1:** The demographic characters of patients with PSIL

	DLBCL	indolent B-cell lymphoma	*p* value	DLBCL	T-cell lymphoma	*p* value
Female (n, %)	4 (40.0)	7 (63.6)	0.395	4 (40.0)	3 (33.3)	1.000
BMI	23.0(20.0–25.5)	20.6(19.0–22.6)	0.122	23.0(20.0–25.5)	18.5(17.4–19.8)	**0.004**
Age of onset	57(46–64)	52(39–66)	0.847	57(46–64)	42(30–56)	**0.023**
Age of examination	59(48–65)	58(41–67)	0.797	59(48–65)	42(30–56))	**0.008**
Cigarette smoking (%)	2 (20.0)	3 (27.3)	1.000	2 (20.0)	3 (33.3)	0.617
Alcohol Drinking (%)	2 (20.0)	0 (0.0)	0.476	2 (20.0)	1 (11.1)	1.000
Family history of tumor (%)	2 (20.0)	0 (0.0)	0.476	2 (20.0)	2 (22.2)	1.000

### DBE system and procedure

Double-balloon endoscope (EN-450 P5/20, Fujifilm, China) was used to evaluate small intestine. All 30 PSIL DBE procedures were conducted by two experienced endoscopists with over 10 years’ experience of DBE practice (Lin Li and Hong Zhang). All the DBE images were reviewed by these two endoscopists as well. For markings from the two endoscopists that did not agree, each endoscopist re-read the cases independently to assess the discordant markings.

No special preparation was required for the antegrade approach besides an 8–12 h fasting. For the retrograde approach, bowel preparation was performed as in colonoscopy. The approach was eventually determined by the endoscopist based on the clinical symptoms or the suspected location of lesions detected by other diagnostic tools. In some circumstances, small bowel lesions were marked with titanium clip to serve as a reference for docking of antegrade or retrograde DBE, consequent endoscopic therapy, or future surgery.

Longitudinal ulcer was defined as ulcer shaped parallel to the long axis of the intestine. Ring-shaped ulcer was defined as ulcer shaped like round disk. Irregular ulcer was defined as irregular shape of the ulcer which cannot be classified in longitudinal ulcer or round ulcer. Deep ulcer was defined as ulcer penetrated through the submucosa into the deeper layers of the gastrointestinal tract. Concomitant colonic ulcer was defined as conic ulcer detected along with small intestinal ulcer in the same patient during examination. Moss cover was defined as ulcer covered with dirty and thick moss. Stenosis was defined as luminal diameter reduction by at least 50% or inability to pass intestinal lumen through the narrowed area without prior endoscopic dilation and with a reasonable amount of pressure applied. Villous atrophy was defined as loss of villous structure with the presences of non-ulcerated, orthogonally converging fissures of the small bowel mucosa [16–18].

### Statistical analysis

The SPSS 27.0 software was used for statistical analysis. Count data were expressed as a percentage, and measurement data were expressed as the median with interquartile range. Differences were evaluated with the *χ*^*2*^ tests and nonparametric tests. We used Fisher’s exact probability when the theoretical frequency was less than five. Pairwise comparisons were conducted to compare the DLBCL group with the T cell lymphoma and B cell lymphoma group respectively. Multiple comparisons were adjusted by the Bonferroni correction (all *p* values < 0.025 was considered to be statistically significant; *p* value corrected for 2 multiple comparisons). Cox regression analysis was performed to assess the significance of various variables for survival.

## Results

### Pathological type of intestinal lymphoma

From May 2013 to September 2021, 255 patients with small intestinal biopsy were included in our study. Among them, 30 were diagnosed with SIL, including 10 (33.3%) with diffuse large B-cell lymphoma (DLBCL), 11 (21.3%) with indolent B cell lymphoma (7 follicular lymphoma (FL)), 4 (12.1%) with mucosa-associated lymphoid tissue lymphoma (MALT), 9 (27.3%) with T-cell lymphoma (5 PTCL, 3 NK/T and 1 MEITL).

### Demographic data

Among all the patients with PSIL, female accounts for 50.0%. The median body mass index (BMI) of patients with TCL is 18.5 (17.4–19.8) kg/m^2^, which is significantly lower than that of patients with DLBCL (*p* = 0.004). However, the weight and BMI of DLBCL patients were similar to that of indolent B-cell lymphoma patients. The median onset and examination age of patients with TCL was 42 (30–56) years, about 10 years earlier than the DLBCL group (*p* = 0.023 and 0.008, respectively). No significant difference was observed in gender, blood type, education level, smoking history, alcohol history, and family history of tumor among the prevalence of different strains of lymphoma (*p* > 0.025, respectively) (Table 1).

### Clinical data

In general, the clinical presentation of intestinal lymphoma is non-specific. The most common symptoms of DLBCL patients were fecal occult blood (70.0%) and weight loss (60.0%). However, most patients diagnosed with indolent B-cell lymphoma and T-cell lymphoma complained abdominal pain (72.7% and 66.7%, respectively). Notably, 54.5% patients with indolent B-cell lymphoma presented constipation. However, there was no significant difference between each group (Table 2).

**Table 2:** The Clinical manifestations of patients with PSIL

	DLBCL	indolent B-cell lymphoma	*p* value	DLBCL	T-cell lymphoma	*p* value
Abdominal pain (%)	4 (40.0)	8 (72.7)	0.198	4 (40.0)	6 (66.7)	0.370
Abdominal distension (%)	3 (30.0)	5 (45.5)	0.659	3 (30.0)	0 (0.0)	0.094
Diarrhea (%)	0 (0.0)	2 (18.2)	0.476	0 (0.0)	2 (22.2)	0.189
Abdominal discomfort (%)	0 (0.0)	3 (27.3)	0.214	0 (0.0)	1 (11.1)	0.450
Nausea and vomiting (%)	0 (0.0)	4 (36.4)	0.090	0 (0.0)	2 (22.2)	0.189
Intestinal obstruction (%)	2 (20.0)	3 (27.3)	1.000	2 (20.0)	0 (0.0)	0.479
Fever (%)	2 (20.0)	2 (18.2)	1.000	2 (20.0)	0 (0.0)	0.479
Night sweats (%)	2 (20.0)	2 (18.2)	1.000	2 (20.0)	0 (0.0)	0.479
Weight loss (%)	6 (60.0)	5 (45.5)	0.230	6 (60.0)	0 (0.0)	0.554
Fatigue (%)	2 (20.0)	2 (18.2)	1.000	2 (20.0)	0 (0.0)	0.479
B symptom	7 (70.0)	5 (45.5)	0.387	7 (70.0)	5 (55.6)	0.650
Melena (%)	3 (30.0)	4 (36.4)	1.000	3 (30.0)	0 (0.0)	0.218
Bloody stools (%)	1 (10.0)	0 (0.0)	0.476	1 (10.0)	1 (11.1)	1.000
Constipation (%)	4 (40.0)	6 (54.5)	0.670	4 (40.0)	1 (11.1)	0.319
Reduced exhaust defecation (%)	1 (10.0)	5 (45.5)	0.149	1 (10.0)	0 (0.0)	1.000
Fecal occult blood (%)	7 (70.0)	5 (45.5)	0.670	7 (70.0)	3 (33.3)	0.370

In laboratory tests, the percentage of elevated C-reactive protein (CRP) and lactate dehydrogenase (LDH) was significantly greater in DLBCL group compared to the indolent BCL group (*p* = 0.004 and *p* = 0.006, respectively). Significant differences were also observed in coagulation tests between DLBCL group and indolent BCL group, such as fibrinogen (FIB) (*p* = 0.013) and D-Dimer (*p* = 0.006). No significant differences were found for the remaining laboratory tests (Table 3).

**Table 3:** The laboratory examination of patients with PSIL

	DLBCL	indolent BCL	*p* value	DLBCL	TCL	*p* value
WBC	5.0(3.8–6.3)	4.2(3.8–5.5)	0.365	5.0(3.8–6.3)	6.4(4.6–7.4)	0.230
Hgb	102.0(93.0–119.3)	107.0(84.3–129.5)	0.847	102.0(93.0–119.3)	115.0(100.0–140.0)	0.331
PLT	259.0(229.8–292.8)	201.0(162.3–290.5)	0.116	259.0(229.8–292.8)	238.0(190.5–318.5)	0.882
NEU	67.5(60.3–74.4)	65.2(48.8–75.3)	0.401	67.5(60.3–74.4)	67.6(58.6–75.4)	0.882
LYM	20.6(13.6–23.8)	23.1(15.5–38.1)	0.270	20.6(13.6–23.8)	17.0(10.5–27.4)	0.412
MONO	8.2(6.8–11.4)	7.6(6.1–12.9)	0.699	8.2(6.8–11.4)	9.9(7.8–14.2)	0.175
CRP	7.0(5.4–42.2)	0.8(0.1–3.9)	**0.004**	7.0(5.4–42.2)	11.5(3.7–73.2)	0.456
ESR	13.0(6.0–39.5)	9.0(2.3–19.0)	0.515	13.0(6.0–39.5)	35.0(10.5–43.5)	0.315
ALB	34.1(32.9–35.3)	40.8(38.1–44.0)	0.116	34.1(32.9–35.3)	39.5(32.2–41.5)	0.941
LDH	256.5(201.5–525.0)	180.5(166.8–213.5)	**0.006**	256.5(201.5–525.0)	185.0(155.0–255.0)	0.113
β2- microglobulin	2334.5(2124.0–2597.5)	1615.0(1177.5–2465.0)	0.556	2334.5(2124.0–2597.5)	3120.0(2034.5–3905.0)	0.730
Cr	78.5(55.8–90.8)	79.8(57.0–88.1)	0.847	78.5(55.8–90.8)	76.8(59.9–117.4)	0.603
UA	285.5(199.0–363.0)	223.5(189.0–268.5)	0.949	285.5(199.0–363.0)	285.0(180.0–335.5)	0.824
FIB	3.8(3.1–5.1)	2.8(2.4–3.9)	**0.006**	3.8(3.1–5.1)	2.9(2.4–4.9)	0.710
D-Dimer	612.0(372.8–1063.5)	294.0(222.0–545.3)	**0.002**	612.0(372.8–1063.5)	2000.0(529.5–4263.5)	0.370
CEA	1.7(0.8–1.9)	1.4(0.8–1.9)	0.605	1.7(0.8–1.9)	2.2(1.5–2.6)	0.035
CA125	19.6(14.0–34.6)	12.3(7.0–19.0)	0.300	19.6(14.0–34.6)	25.7(23.1–63.3)	0.035
TSPOT(+ , %)	2 (28.6)	3 (33.3)	1.000	2 (28.6)	0 (0.0)	0.175
CMV (+ , %)	0 (0.0)	0 (0.0)	—	0 (0.0)	0 (0.0)	—
EBV (+ , %)	1 (20.0)	2 (22.2)	1.000	1 (20.0)	3 (60.0)	0.524

### Imaging findings

**Fig. 1:**
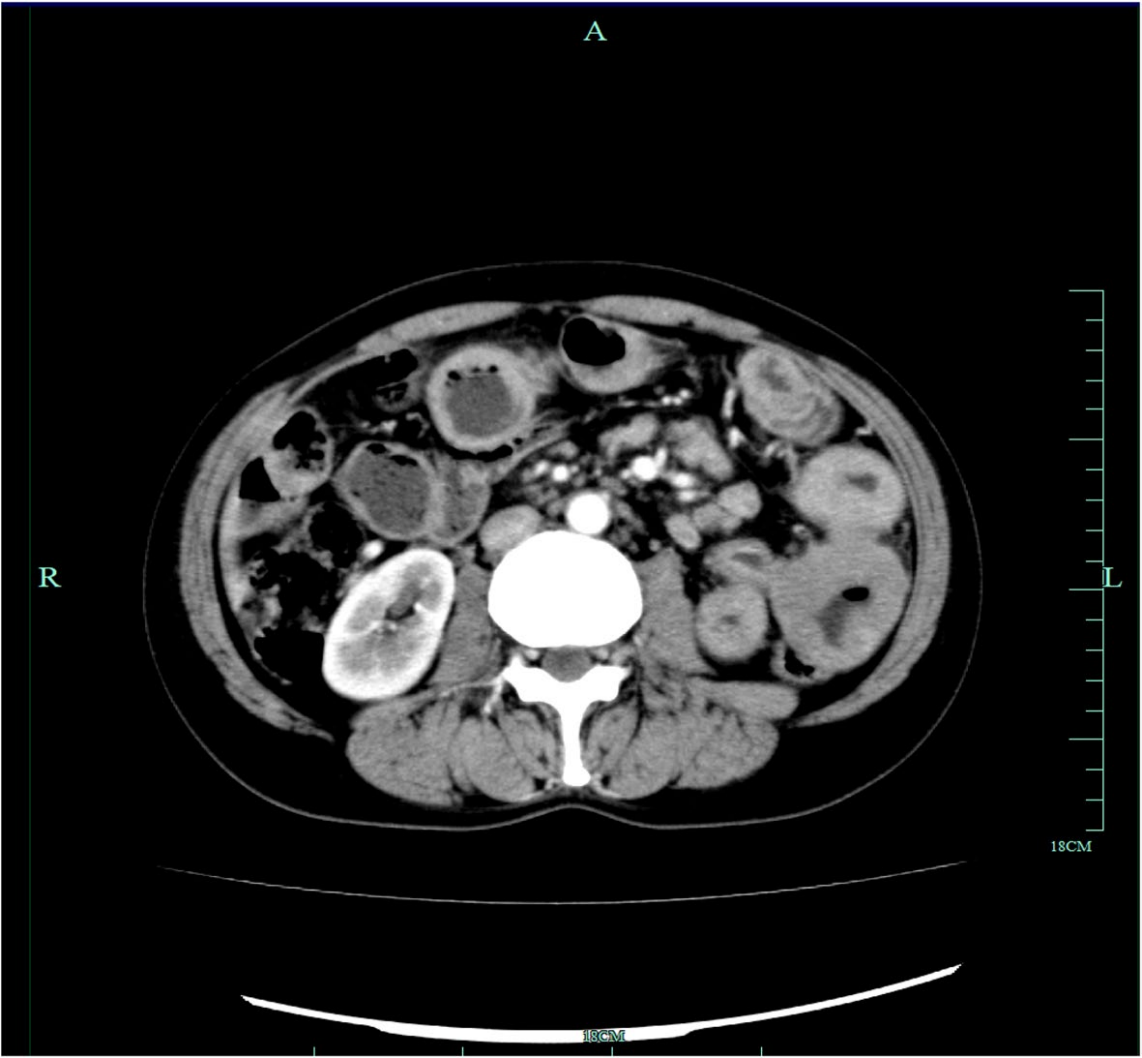
CT enterography showing the aneurysmal dilation

The imaging data were showed in Table 4. Indolent BCL patients showed shorter length of lesion segments (*p* = 0.034), while DLBCL patients and TCL patients were more likely to have longer lesion segment. Intestinal wall thickness of DLBCL patients were more frequent than indolent BCL patients (*p* = 0.003). Additionally, compared to patients with indolent BCL, patients with DLBCL were more prone to aneurysmal dilation (*p* = 0.020). Aneurysmal dilatation radiologically presents as segmental focal dilation of the small intestine with thickening of the bowel wall at the site of dilation, secondary to malignant infiltration (Fig. 1). Loss of bowel tone occurs due to penetration of the muscularis propria as well as destruction of the autonomic nerve plexus. It was first described in 1969 by Cupps et al., who noted the aneurysmal pattern in 35% of small bowel lymphoma cases on fluoroscopic barium studies[19].

**Table 4:** The CT findings of patients with PSIL

	DLBCL	indolent BCL	*p* value	DLBCL	TCL	*p* value
Lesion segment length (cm)	12.0(7.3–18.3)	6.0(1.0–8.8)	0.034	12.0(7.3–18.3)	8.0(4.5–16.5)	0.491
Intestinal wall thickening	10 (100.0)	8 (72.7)	0.214	10(100.0)	8(88.9)	1.000
Intestinal wall thickness (cm)	1.6(1.2–2.9)	0.9(0.2–1.2)	**0.003**	1.6(1.2–2.9)	1.2(1.1–1.4)	0.029
Intestinal lumen dilation	5 (50.0)	3 (27.3)	0.387	5 (50.0)	2(22.2)	1.000
Intestinal lumen stenosis	4 (40.0)	3 (27.3)	0.659	4 (40.0)	0(0.0)	0.206
Aneurysmal dilation	8 (88.9)	3 (30.0)	**0.020**	8 (88.9)	2 (33.3)	0.089
Enlarged lymph nodes	9 (90.0)	11 (100.0)	0.476	9 (90.0)	8(88.9)	1.000

### DBE findings

The DBE findings are showed in Table 5. The most frequently involved site in the indolent BCL and TCL patients was the jejunum (63.6% and 66.7%, respectively). But in DLBCL patients, the ileum was more frequently involved (70.0%). Most of the lesions presented as ulcers, there was no significant difference between the three groups. In terms of ulcer morphology, patients with DLBCL had significantly higher proportion of deep ulcers than indolent BCL, with no difference compared with TCL (*p* = 0.009). Few indolent BCL patients (18.2%) presented as irregular, large and deep ulcers.

**Table 5:** The DBE findings of patients with PSIL

	DLBCL	indolent B-cell lymphoma	*p* value	DLBCL	T-cell lymphoma	*p* value
**Lesion sites**						
Duodenum (n, %)	0 (0.0)	2 (18.2)	0.476	0 (0.0)	3 (33.3)	0.087
Jejunum (n, %)	4 (40.0)	7 (63.6)	0.395	4 (40.0)	6 (66.7)	0.370
Ileum (n, %)	7 (70.0)	5 (45.5)	0.387	7 (70.0)	4 (44.4)	0.370
Ileocecal valve involved (n, %)	0 (0.0)	0 (0.0)	—	0 (0.0)	1 (11.1)	0.474
**Ulcers (n, %)**	10 (90.9)	9(81.8)	1.000	10 (90.9)	8 (88.9)	1.000
Longitudinal ulcers (n, %)	0 (0.0)	0 (0.0)	—	0 (0.0)	1 (11.1)	0.474
Round ulcers (n, %)	3 (30.0)	5 (45.5)	0.659	3 (30.0)	3 (33.3)	1.000
Irregular ulcers (n, %)	7 (70.0)	2 (18.2)	0.030	7 (70.0)	4 (44.4)	1.000
Deep ulcers# (n, %)	8 (80.0)	2 (18.2)	**0.009**	8 (80.0)	6 (66.7)	0.628
Concomitant colonic ulcers (n, %)	1 (10.0)	0 (0.0)	0.476	1 (10.0)	0 (0.0)	1.000
Moss covered (n, %)	7 (70.0)	3 (23.3)	0.086	7 (70.0)	7 (77.8)	1.000
Stenosis (n, %)	3 (30.0)	4 (36.4)	1.000	3 (30.0)	3 (33.3)	1.000
Villous atrophy (n, %)	0 (0.0)	1 (9.1)	1.000	0 (0.0)	1 (11.1)	0.474

# “Deep ulcers” refers to a type of ulcer that penetrates through the submucosa into the deeper layers of the gastrointestinal tract (Fig. 2) [20]

**Fig. 2:**
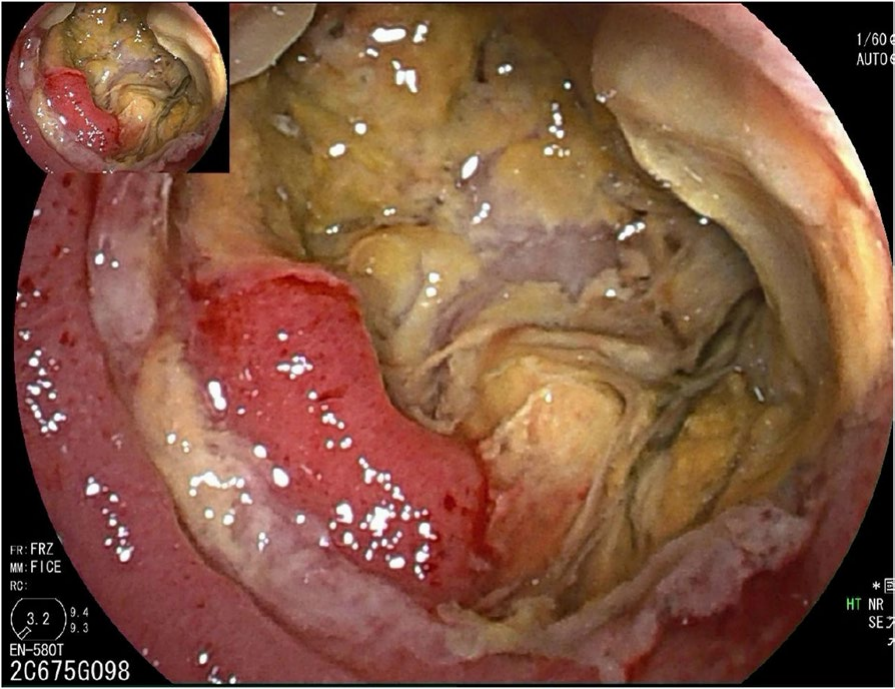
DBE image showing the deep ulcer

### Survival analyses

A total number of 30 cases were included in survival analysis. The median follows up period was 21.5 months. Seven cases died during follow up. Since the infrequency of the outcome variables occurrence, survival analyses were performed using univariable Cox regression analysis (Table 6). The Cox regression analysis of overall survival yielded a hazard ratio of 13.77 (95% *CI*, 1.22–154.91, *p* = 0.034) for drink, a hazard ratio of 10.50 (95%*CI*, 1.08–102.46, *p* = 0.043) for LDH, and a hazard ratio of 13.73 (95%*CI*, 1.22–154.91, *p* = 0.034) for concomitant colonic ulcers. Besides, we used Cox regression to estimate the difference in prognosis among three PSIL subtypes. No significant difference was found among the three PSIL subtypes.

**Table 6:** The result of univariate Cox regression

Variable	HR	95%CI	*p* value
Sex	2.68	0.27–25.84	0.391
BMI	1.06	0.72–1.57	0.746
Age	0.99	0.91–1.08	0.941
Alcohol Drinking	13.72	1.21–154.90	**0.034**
LDH	10.50	1.07–102.45	**0.043**
Aneurysmal dilation	1.07	0.15–7.60	0.945
Intestinal wall thickness	1.85	0.49–7.03	0.360
Deep ulcers	2.68	0.27–25.84	0.391
Concomitant colonic ulcers	13.72	1.21–154.90	**0.034**
Stenosis	0.53	0.05–5.15	0.589

## Discussion

The traditional approach classifying lymphomas into Hodgkin's and non-Hodgkin's has been widely used in hematological researches. However, Hodgkin's lymphoma is an uncommon type in small intestinal lymphoma [21, 22]. Moreover, in our case series, there was no patient diagnosed with Hodgkin lymphoma. Therefore, it is inappropriate to make Hodgkin's lymphoma as a universal reference. Meanwhile, DLBCL has been reported to be the most common subtype in small intestinal lymphoma [2]. The clinical manifestations and differential diagnosis of indolent B and T cells in small intestinal lymphoma have been rarely mentioned in previous literature. Therefore, we proposed this rational of comparison in our study to provide more information for clinical practice.

In our study, the most common small intestinal lymphomas are of B cell lineage, with a third of T-cell lymphoma which is in accordance with previous researches [23, 24]. The proportion of the DLBCL (33.3%) was similar to other research [25]. The evaluation, diagnosis, treatment and prognosis of this disease are different from different strains of lymphomas and other GI cancers, so it is important to recognize it properly. However, most patients have overlapping symptoms of other gastrointestinal diseases, including abdominal pain, abdominal distension, diarrhea, nausea and vomiting, gastrointestinal bleeding, intestinal obstruction or perforation, and palpable abdominal masses, as literature reported [26, 27]. In our series, more than a half of the patients exhibit systemic symptoms, such as B symptom (fever, night sweat and/or weight loss). As previous series reported, Ileum is the most common site (60%-65%) involving small intestine lymphoma followed by jejunum (20%-25%), duodenum (6%-8%) and other sites (8%-9%). In our study, Ileum is the most common site in DLBCL patients (70%), while in indolent BCL and TCL patients, jejunum is the most common involving site (over 60%). The age of onset of lymphomas varies depending on the histologic subtype. Notably, the onset and examination age of TCL patients is significantly earlier than DLBCL patients [28, 29]. Consistent with previous studies, most TCL patients have weight loss and the BMI of patients are also significantly decreased [30, 31].

Regarding the laboratory examination, CRP and LDH levels elevated significantly in patients with DLBCL rather than indolent BCL. The inflammatory tumor microenvironment has been under focus in the recent years and inflammatory status is essential in tumor microenvironment during lymphomagenesis [32, 33]. The elevation of CRP in DLBCL may result from higher disease activity, deeper and larger ulcer or occult infection. LDH is also considered as an important factor in different prognostic scoring systems [34], which works more efficiently in B-cell lymphomas, especially DLBCL [33]. Wu, et al. reported that CRP and LDH were significantly higher in patients with DLBCL than in follicular lymphoma (a predominant type of indolent BCL in our case series) [35]. However, the role of these serum markers in differentiating PSIL remains unclear. Our results indicate that the elevation of CRP and LDH might be helpful in differentiating DLBCL from indolent BCL. Further prospective studies with larger sample size are needed to confirm it.

Patients with PSIL are more often diagnosed at endoscopy for non-specific gastrointestinal symptoms. Diagnostic evaluation of suspected small bowel lymphoma includes contrast-enhanced CT, PET, conventional endoscopy, and capsule endoscopy [27, 36]. CT and/or contrast imaging are usually the initial diagnostic tools, although more patients are definitively diagnosed by DBE [3, 37]. According to the CT image, patients with DLBCL have longer intestinal lesions, thicker intestinal walls, and more aneurysmal dilation. In a small number of patients, the diagnosis can’t be confirmed before the serious complications occur, like obstruction, perforation, or hemorrhage and emergency surgery with resection of the diseased bowel segment. In our study, DLBCL, as an aggressive lymphoma, is more likely to appear as large, deep and irregular ulcers under endoscopy. Notably, there is a patient with monomorphic epitheliotropic intestinal T-cell lymphoma (MEITL). The DBE showed a diffuse edematous mucosa, erosion, and ulcerative tumors. A case series by Ishibashi, H. et al. reported the endoscopic findings of MEITL patients included circumferential ulcerated tumors and an edematous and granular mucosa, with a nodular or mosaic pattern, in the duodenum, jejunum, and ileum [38]. We still need more cases to validate this result. Besides, 12(40.0%) patients showed no signs of tumor specific in endoscopy biopsy and were finally diagnosed by surgery biopsy, 3 of which underwent emergency surgery for intestinal perforation, gastrointestinal bleeding and intestinal obstruction. Possible reasons are as follows: (1) The lamina propria of mucosa in lesion site is soft and easy to be damaged during biopsy, the location of autolysis sample is inaccurate and the depth is not enough; (2) The glandular duct tissue was not collected, which make it hard to distinguish from poorly differentiated adenocarcinoma; (3) Early lesions were located in submucosa and infiltrated downward, while the mucosa was intact and easy to overlook under endoscope. And the insufficient biopsy depth may also cause missed diagnoses. The lesion of aggressive lymphoma is relatively deep, so multiple, multi-part, deep chiseled samples are required for biopsy, and a negative biopsy cannot exclude the disease. Surgical biopsy may be considered when multiple endoscopy biopsies are negative, or the disease progresses rapidly.

Regarding treatment approach of PSIL, there are many options including surgical resection, chemotherapy, and radiotherapy. The intestine is less suitable for radiotherapy than the stomach, so in our hospital patients with PSIL were rarely treated with radiotherapy. A retrospective analysis of 27 cases of primary intestinal lymphoma by Khosla et al [39]. showed that OS was up to 79.50% in the patients with surgery plus chemotherapy for the past 5 years, and was significantly better than that of the patients with chemotherapy alone (13.9%). In our study, the survival rate of patients who received chemotherapy alone was 41.7% (Chemotherapy is mainly based on R-CHOP regimens). The survival rate after surgery alone is 60.0%, while the survival rate after surgery combined with systemic chemotherapy turned out to be 87.5%. These findings suggest that even with complete resection, patients who received systemic chemotherapy may achieve a better prognosis.

The prognosis of intestinal lymphoma is closely related mainly to the disease staging and IPI score [40]. In our study, the various histological subtypes of lymphocytic lymphomas have not shown to be a major determinant of survival, which is consistent with several reported literature. So early diagnosis and treatment contribute to a favorable outcome[3, 26]. Because of the indefinite and nonspecific nature of patients' complaints, a high level of vigilance for this disease is essential for the early diagnosis and treatment of intestinal lymphoma. Cox regression revealed that concomitant colonic ulcer and LDH are poor prognostic risk factors for PSIL. Serum LDH represents a quantitative measure of tumor burden and aggressiveness in NHL. Concomitant colonic ulcer also represents a multiple sites invasion in PSIL which might correlated with a poor prognostic. However, since the infrequency of the outcome variables in our study, we can only perform univariable Cox regression rather than multiple regression. Further studies with lager sample size are needed to confirm our results.

There are certain limitations in this study. Firstly, the retrospective nature and limited sample size in this study might cause bias. Although PSIL is a rare subtype both in intestinal malignant tumor and lymphoma, a prospective, multicenter study with larger sample size is needed to better address this issue. Besides, this study mostly focused on the clinical and endoscopic presentation with limited information about the treatment and prognose of PSIL. We are looking forward to have collaboration with hematologists for further investigations. Thirdly, the duration and amount of alcohol drinking were not recorded specifically in our research. Further studies are needed to investigate the specific relationship between alcohol consumption and the prognosis of PSIL. Fourthly, we can only perform univariable Cox regression models rather than multiple regression model due to the infrequency of the outcome variables occurrence, which was a significant methodology limitation of our research. Further study with larger sample size and higher frequency of outcome events is needed to verify our results.

## Conclusion

In conclusion, our study summarized the characteristics of different subtypes of small intestinal lymphoma, especially the small intestinal endoscopic features, which provides assistance for the diagnosis and identification of small bowel lymphoma. Compared to patients with indolent BCL, patients with DLBCL were more prone to present as thicker intestinal wall and aneurysmal dilation. In terms of ulcer morphology, patients with DLBCL had significantly higher proportion of deep ulcers than indolent BCL, with no difference compared with TCL. Drink, concomitant colonic ulcers and higher level of LDH are risk factors for mortality in patients with PSIL.

## Data Availability

The datasets used and/or analyzed during the current study are available from the corresponding author (MJ) on reasonable request.
